# Diagnosis of urinary bladder diseases in dogs by using two-dimensional and three-dimensional ultrasonography

**DOI:** 10.14202/vetworld.2015.819-822

**Published:** 2015-07-07

**Authors:** Dehmiwal Dinesh, S.M. Behl, Prem Singh, Rishi Tayal, Madan Pal, R.K. Chandolia

**Affiliations:** 1 Department of Veterinary Surgery and Radiology, Lala Lajpat Rai University of Veterinary & Animal Sciences, Hisar, Haryana, India; 2 Department of Veterinary Gynaecology and Obstetrics, Lala Lajpat Rai University of Veterinary & Animal Sciences, Hisar, Haryana, India

**Keywords:** anechoic, cystitis, cystoliths, hyperechoic, hypoechoic, neoplasia, ultrasonography

## Abstract

**Aim:**

The objective of this study was to obtain and compare two-dimensional (2D) and three-dimensional (3D) ultrasonographic images of the urinary bladder in different disease conditions.

**Materials and Methods:**

The present study was conducting on total 10clinical cases of the urinary bladder in dogs. The ultrasound (US) machine used for this study was 3D US machine (Nemio-XG: Toshiba, Japan) having a four-dimensional volumetric probe.

**Results:**

In the present study, the inflamed thickened wall was clearly visible with the distinction of different layers of the urinary bladder wall in some of the cases of cystitis using 2D ultrasonography. In 3D sonogram, the urinary bladder was visualized as a large anechoic structure with no distinction of different layers of the bladder wall. The cystoliths were clearly visible as hyperechoic structures with distal acoustic shadow in 2D sonogram and appeared as a bright echogenic area in 3D sonogram. In case of urinary bladder neoplasia in 2D ultrasonogram, the bladder lumen was found to be occluded with a large growth imaged as focal anechoic areas in the tissue of mixed echogenicity with small hyperechoic dots in this tissue parenchyma. In 3D ultrasonogram, a tissue of mixed echogenicity of pus was also observed.

**Conclusion:**

From the present study it was concluded that 2D and 3D ultrasonography is very helpful for diagnosis of different clinical conditions of the urinary bladder such as cystitis, cystoliths, and urinary bladder neoplasia. The cavity of urinary bladder was more clearly visualized in 3D ultrasonography, but the distinction of different layers of the bladder wall was visualized only in 2D ultrasonography. The distinct shadow of pus and cystoliths were visible in 2D ultrasonogram. The visualization of pus in 3D ultrasonography was done for the first time in present study.

## Introduction

The urinary system plays an important role in excretion of the waste products and maintenance of electrolyte balance. Any pathology of the urinary system can cause metabolic disturbances and derangements of fluid, electrolyte, and acid-base balance. Ultrasound (US) is the most commonly used imaging method for studying urinary tract disorders in dogs, as it is easy to perform, inexpensive and provides excellent contrast resolution in real-time [1]. US is furthermore useful for guiding interventional procedures [2]. The urinary bladder is ideally suited for sonographic examination because of its superficial position and its fluid content so that little attenuation of the sound beam occurs. It may have various abnormalities such as cystic calculi [3], cystic neoplasm and cystitis [4]. Urinary tract infection is common in dogs; especially in females is most often happening the result of ascending fecal bacterial contamination of the vulva, perivulvar skin, vestibule [5]. Abnormalities of urinary bladder either in its wall or in the density of urine can be judged by ultrasonography [6].

Ultrasonography is a more sensitive technique for detection of gas within the urinary bladder at an early stage of emphysematous cystitis, and the condition may be underestimated if only radiographs are made [4]. The conditions of urinary bladder especially infection of the urinary tract can be diagnosed by microscopic and culture sensitivity test. However, the conditions of cystoliths and cystic neoplasm cannot be diagnosed by urine examination alone. Renal calculi are a common problem in dogs. The radiography can be used to diagnose the conditions of cystic calculi, but the diagnosis becomes challenging if urinary stones are radiolucent [7]. The urinary calculi can also be easily diagnosed with ultrasonography. The hyperechoic structure showing acoustic shadowing below it is a confirmation of calculi.

The three-dimensional (3D) ultrasonography, available in medicine for over 10years enabled great advances in the area of diagnostic imaging. This technique facilitates the volumetric study of organs and structures, besides it, provide a third image plane, the coronal plane, which allows more precisely volumetric calculation mainly those of irregular shapes. The 3D ultrasonography is still a new technique in veterinary medicine, and few are the scientific papers that report its experimental use [8].

## Materials and Methods

### Ethical approval

The study was conducted after the approval of the Institutional Animal Ethics Committee.

### Study area

The study was conducted in the Department of Veterinary Surgery and Radiology with collaboration of Department of Veterinary Gynaecology and Obstetrics, College of Veterinary Sciences, Lala Lajpat Rai University and Animal Sciences (LUVAS), Hisar (Haryana).

### Animals

The dogs (n=10) with pathological conditions of the urinary bladder reported to Teaching Veterinary Clinical Complex (TVCC) College of Veterinary Sciences, Lala Lajpat Rai University and Animal Sciences, Hisar (Haryana) were used for the study.

### Ultrasonographic examination

The study was conducted on 10 clinical cases of different age groups of dogs suffering from urinary bladder diseases brought to TVCC, LUVAS, Hisar. The dogs were sedated with xylazine @ 1mg/kg body weight for restraining. For scanning urinary bladder they were positioned in dorsal and lateral recumbency. A curvilinear probe was placed just cranial to the pelvic inlet and perpendicular to the skin in the ventral midline in female and right or left side of the prepuce in males. Normally the bladder does not extend beyond umbilicus. The urinary bladder was observed as anechoic structure surrounded by a smooth regular outline of an echo dense wall. Scanning area was shaved properly and enough gel was applied over the site and the surface of the transducer to get a better image. The US machine used for this study was 3D US machine (Nemio-XG: Toshiba, Japan) having four-dimensional (4D) volumetric probe. The images were acquired with 3-6 MHz two-dimensional (2D) curvilinear transducer and 4.2-6 MHz 4D volumetric curvilinear transducer.

## Results

### Cystitis

Total five cases of cystitis were brought to the TVCC, Hisar. In the first case, a dog was reported with the history of urine incontinence, black feces, stretching of legs and vomition, dribbling of saliva. In abdominal radiograph, nothing abnormal was detected. In 2D ultrasonogram (Figure-1a) thick and distinct three layered urinary bladder wall was visualized as submucosa and serosa appeared hyperechoic with hypoechoic muscularis layer in between them. In 3D ultrasonogram, (Figure-1b) hyperechoic thick wall of the bladder with no distinction of three layers was imaged. In the second case of cystitis, in 2D ultrasonogram the bladder wall appeared thicker without distinction of different layers in the anechoic lumen of the bladder. In 3D ultrasonogram, the bladder wall thickness was not visualised in the anechoic lumen. In a third case in 2D ultrasonogram, the urinary bladder appeared as a large spheroid with an anechoic lumen having a thick hyperechoic wall. The bladder wall was uniformly thick, and distinction of different layers was absent. In 3D ultrasonogram, the different layers of bladder wall were clearly visualized. In the fourth case, in 2D ultrasonogram, the bladder wall was thicker than normal indicative of cystitis. In 3D ultrasonogram, the urinary bladder was visualized as a large anechoic structure with no distinction of different layers. In the fifth case in 2D ultrasonogram, the urinary bladder appeared as a small anechoic structure. The bladder wall was thicker than normal indicative of cystitis. In 3D ultrasonogram, the urinary bladder was visualized as a small anechoic structure. The wall of urinary bladder was thick and hyperechoic with no distinction of different layers.

**Figure-l:**
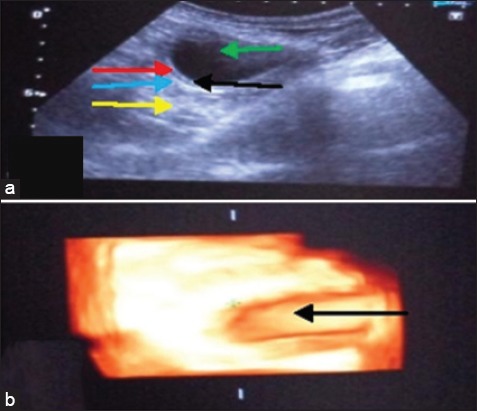
Urinary bladder with cystitis (a) In two-dimensional the bladder lumen (green arrow), acoustic enhancement (yellow arrow) distally, thickened bladder wall with clear distinction of submucosa (black arrow), muscularis (red arrow) and serosa (blue arrow). (b) In three-dimensionalthe bladder lumen (black arrow) is visible only without further details.

### Cystoliths

**Figure-2:**
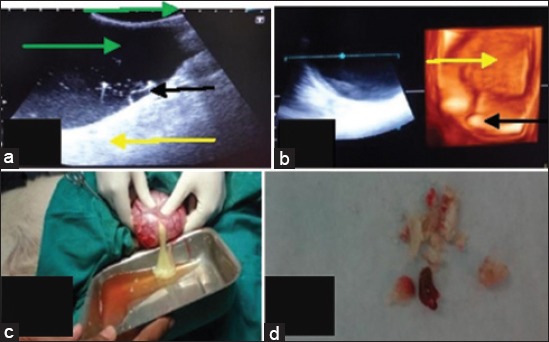
Urinary bladder with cystoliths (a) In two-dimensionalthe bladder lumen (green arrow), strong acoustic enhancement (yellow arrow) distally, fluctuating pus flakes (black arrow) and echogenic calculi (red arrow). (b) In three-dimensional the bladder lumen (yellow arrow) is visible having echogenic calculi (black arrow) in it. (c) It was pus in the dependent portion of the bladder which appears as hypoechoic area. (d) Cystoliths removed after cystotomy.

Cystoliths were diagnosed in four dogs brought to the TVCC, Hisar. In the first case, a dog of 6 years age was brought with the history of urine retention with a distended abdomen. In 2D ultrasonogram, at the dependent part of the bladder there were many hyperechoic rounded calculi were seen in the anechoic lumen of the bladder. Strong acoustic enhancement distal to the bladder also contributed to less distinction of the bladder wall. In 3D ultrasonogram, cystoliths were appeared collectively as a bright echogenic area in the anechoic lumen of the urinary bladder. The cystoliths were removed after cystotomy. In the second case, a dog of 8 years age was brought with the history of urine incontinence for and constipation. The laboratory values of BUN and creatinine were 152mg/dl and 13.3mg/dl. In 2D ultrasonogram (Figure-2a) the bladder lumen was half hypoechoic due to pus in the dependent portion and calculi appeared as bright echogenic structures. Strong acoustic enhancement was present below the urinary bladder. In 3D ultrasonogram (Figure-2b) also two areas of different echogenicity were visualized in the bladder lumen. The calculi appeared as bright echogenic structures. After cystotomy pus and cystoliths were removed (Figure-2c and d). In a third casein 2D ultrasonogram, the urinary bladder appeared as an anechoic structure with hyperechoic calculi in the dependent part. There was strong acoustic shadow below these calculi. In 3D ultrasonogram, same changes were observed. In the fourth case in 2D ultrasonogram, hyperechoic calculi were seen in anechoic lumen of bladder. There was strong acoustic shadow below these calculi. In 3D ultrasonogram, calculi were imaged as bright echogenic structures with thickened wall of urinary bladder.

### Urinary bladder neoplasia

**Figure-3:**
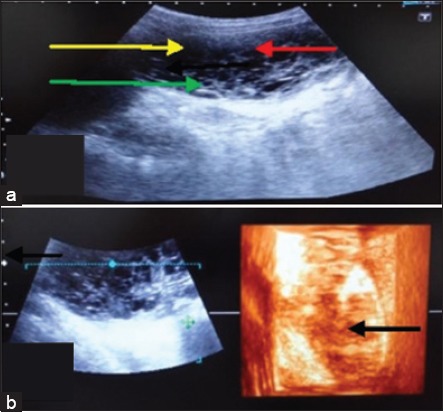
Urinary bladder neoplasia. (a) In two-dimensional the bladder lumen is obliterated by a large growth (yellow arrow), focal anechoic areas (red arrow) and small hperechoic dots (green arrow) representing lumen of blood vessels are also visible in it. (b) In three-dimensional a tissue of mixed echogenicity (black arrow) appears.

A dog of 10years age was brought to the TVCC with the history of scanty urination and anorexia, distended abdomen for the last 1-week. In 2D ultrasonogram (Figure-3a) the bladder lumen was found to be occluded with a large growth having focal anechoic areas with small hyperechoic dots in this tissue parenchyma. In 3D ultrasonogram (Figure-3b) a tissue of mixed echogenicity of patchy hypoechoic and hyperechoic areas was seen together. The demarcation of the bladder wall was not clear.

## Discussion

The inflammation of the urinary bladder is a common clinical problem in animals. Retention of urine is the most predisposing factor for cystitis [9]. Two cases of cystitis were observed with retention of urine due to calculi and prostate enlargement respectively. They also reported that due to the short urethra the female animals of all species are more prone to the ascending infection of the urinary tract. In the five cases of cystitis two animals were female in the study. In other two cases, descending infection may be there as nephritis was also observed in them. In all the five cases, increased thickness of bladder wall was the common ultrasonographic finding. Similar findings were observed by Leveille *et al*. [10] and Biller *et al*. [11]. The bladder wall appeared hypoechoic and thickened in all the cases. Maxie [12] reported that these changes may be related to the leukocyte infiltration and haemorrhage in all the layers of the bladder wall. In 3D ultrasonogram, the bladder wall was also appeared thicker than normal in the anechoic lumen of the urinary bladder. Urine culture is another diagnostic method for cystitis, which is expensive and time-consuming and due to the dilution of bacteria during the treatment period, the measurement errors may occur [4].

In this study, four cases of cystoliths were diagnosed by ultrasonography. In 2D ultrasonogram, the calculi appeared as bright hyperechoic round structures in the dependent portion of the urinary bladder except in one case. Acoustic shadowing distal to these calculi was present in all the cases. Similar findings were also reported by Saini and Singh [13], Kundu and Ghosh [3] and Verma *et al*. [14]. Finn-Bodner [15] reported that cystic calculi appeared as curvilinear hyperechoic interfaces blocking much of the sound beam at first fluid-calculus interface and hence causing a distal acoustic shadow. In one of the four cases, pus was seen instead of calculi in the partially anechoic lumen of the urinary bladder. Ultrasonographically, hypoechoic area with some flexible echogenic structures in the anechoic lumen of urinary bladder was imaged. On cystotomy, the bladder was found to be filled with pus. It was pus in the bladder appearing hypoechoic with anechoic urine. In 3D ultrasonogram also two areas of different echogenicity were visualised in the bladder lumen. No previous record of visualisation of pus in 3D ultrasonography is available. Dennis and Hamm [16] reported that pus appeared anechoic to hyperechoic in the cases of pyometra in bitches. The hypoechoic dependent portion and anechoic upper portion were visualised clearly. The calculi were appeared as bright echogenic structures.

One case of urinary bladder neoplasia was also observed in the study. The urinary obstruction is the presenting sign in urinary bladder neoplasia [17]. In 2D ultrasonogram, a large hyperechoic structure was observed with numerous anechoic foci in it. Biller *et al*. [11] also reported that bladder neoplastic masses are echogenic. Small hyperechoic dots were also visualised in this tissue parenchyma. The bladder wall was not distinct. Thus, the sonographic features were suggestive of urinary bladder neoplasia. In 3D ultrasonogram, a tissue of mixed echogenicity was observed. Patchy hypoechoic and hyperechoic areas appeared together. In transitional cell carcinoma use of 3D ultrasonography can provide a less expensive and more practical method for monitoring response to treatment than computed tomography and was more accurate than 2D ultrasonography [18].

## Conclusion

From the present study, it was concluded that 2D and 3D ultrasonography is very helpful for diagnosis of different clinical conditions of the urinary bladder such as cystitis, cystoliths, and urinary bladder neoplasia. The cavity of urinary bladder was more clearly visualised in 3D ultrasonography, but the distinction of different layers of the bladder wall was visualised only in 2D ultrasonography. The distinct shadow of pus and cystoliths were visible in 2D ultrasonogram. The visualisation of pus in 3D ultrasonography was done for the first time in the present study.

## Authors’ Contributions

DD, SMB, and PS have designed the study and planned the research experiment. DD performed the research experiments. PS, RT, and RKC supervised the research. MP helped in conducting experiments. All the authors read and approved the final manuscript.
